# 3-Methyl-*N*-(4-methyl­phen­yl)benzamide

**DOI:** 10.1107/S1600536811040967

**Published:** 2011-10-08

**Authors:** Vinola Z. Rodrigues, Viktor Vrábel, B. Thimme Gowda, Jozef Kožíšek

**Affiliations:** a Department of Chemistry, Mangalore University, Mangalagangotri 574 199, Mangalore, India; b Institute of Physical Chemistry and Chemical Physics, Slovak University of Technology, Radlinského 9, SK-812 37 Bratislava, Slovak Republic

## Abstract

In the title compound, C_15_H_15_NO, the two aromatic rings make a dihedral angle of 70.06 (3)°, while the central amide core –NH—C(=O)– is twisted by 30.24 (4) and 40.16 (3)° out of the planes of the 3-methyphenyl and 4-methyphenyl rings, respectively. The methyl groups are disordered over two equally occupied positions. In the crystal, inter­molecular N—H⋯O hydrogen bonds link the mol­ecules into infinite chains running along the *a* axis.

## Related literature

For the preparation of the title compound, see: Gowda *et al.* (2003 ▶). For studies on the effects of substituents on the structures and other aspects of *N*-(ar­yl)-amides, see: Bowes *et al.* (2003 ▶); Gowda *et al.* (2000 ▶); Saeed *et al.* (2010 ▶), on *N*-(ar­yl)-methane­sulfonamides, see: Gowda *et al.* (2007 ▶), on *N*-(ar­yl)-aryl­sulfonamides, see: Shetty & Gowda (2005 ▶) and on *N*-chloro-aryl­sulfonamides, see: Gowda & Shetty (2004 ▶).
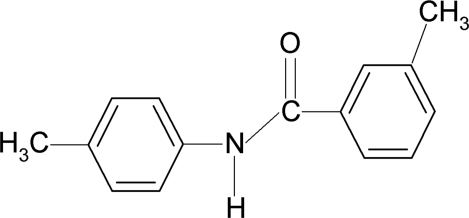


## Experimental

### 

#### Crystal data

C_15_H_15_NO
*M* *_r_* = 225.28
Monoclinic, 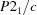

*a* = 5.2694 (2) Å
*b* = 14.0877 (5) Å
*c* = 16.5665 (9) Å
β = 92.588 (4)°
*V* = 1228.54 (9) Å^3^
*Z* = 4
Mo *K*α radiation
μ = 0.08 mm^−1^
*T* = 295 K
0.32 × 0.21 × 0.12 mm

#### Data collection

Oxford Diffraction Xcalibur diffractometer
Absorption correction: multi-scan (*CrysAlis RED*; Oxford Diffraction, 2009 ▶) *T*_min_ = 0.976, *T*_max_ = 0.989
18822 measured reflections
2800 independent reflections
1530 reflections with *I* > 2σ(*I*)
*R*_int_ = 0.037

#### Refinement

*R*[*F* ^2^ > 2σ(*F* ^2^)] = 0.036
*wR*(*F* ^2^) = 0.088
*S* = 0.83
2800 reflections
158 parameters
1 restraint
H atoms treated by a mixture of independent and constrained refinement
Δρ_max_ = 0.13 e Å^−3^
Δρ_min_ = −0.15 e Å^−3^

### 

Data collection: *CrysAlis CCD* (Oxford Diffraction, 2009 ▶); cell refinement: *CrysAlis CCD*; data reduction: *CrysAlis RED* (Oxford Diffraction, 2009 ▶); program(s) used to solve structure: *SHELXS97* (Sheldrick, 2008 ▶); program(s) used to refine structure: *SHELXL97* (Sheldrick, 2008 ▶); molecular graphics: *DIAMOND* (Brandenburg, 2002 ▶); software used to prepare material for publication: *SHELXL97*, *PLATON* (Spek, 2009 ▶) and *WinGX* (Farrugia, 1999 ▶).

## Supplementary Material

Crystal structure: contains datablock(s) I, global. DOI: 10.1107/S1600536811040967/bq2308sup1.cif

Structure factors: contains datablock(s) I. DOI: 10.1107/S1600536811040967/bq2308Isup2.hkl

Supplementary material file. DOI: 10.1107/S1600536811040967/bq2308Isup3.cml

Additional supplementary materials:  crystallographic information; 3D view; checkCIF report

## Figures and Tables

**Table 1:** Hydrogen-bond geometry (Å, °)

*D*—H⋯*A*	*D*—H	H⋯*A*	*D*⋯*A*	*D*—H⋯*A*
N1—H1⋯O1^i^	0.86 (1)	2.30 (1)	3.0910 (13)	155 (1)

Symmetry code: (i) 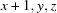.

**Fig. 1.**
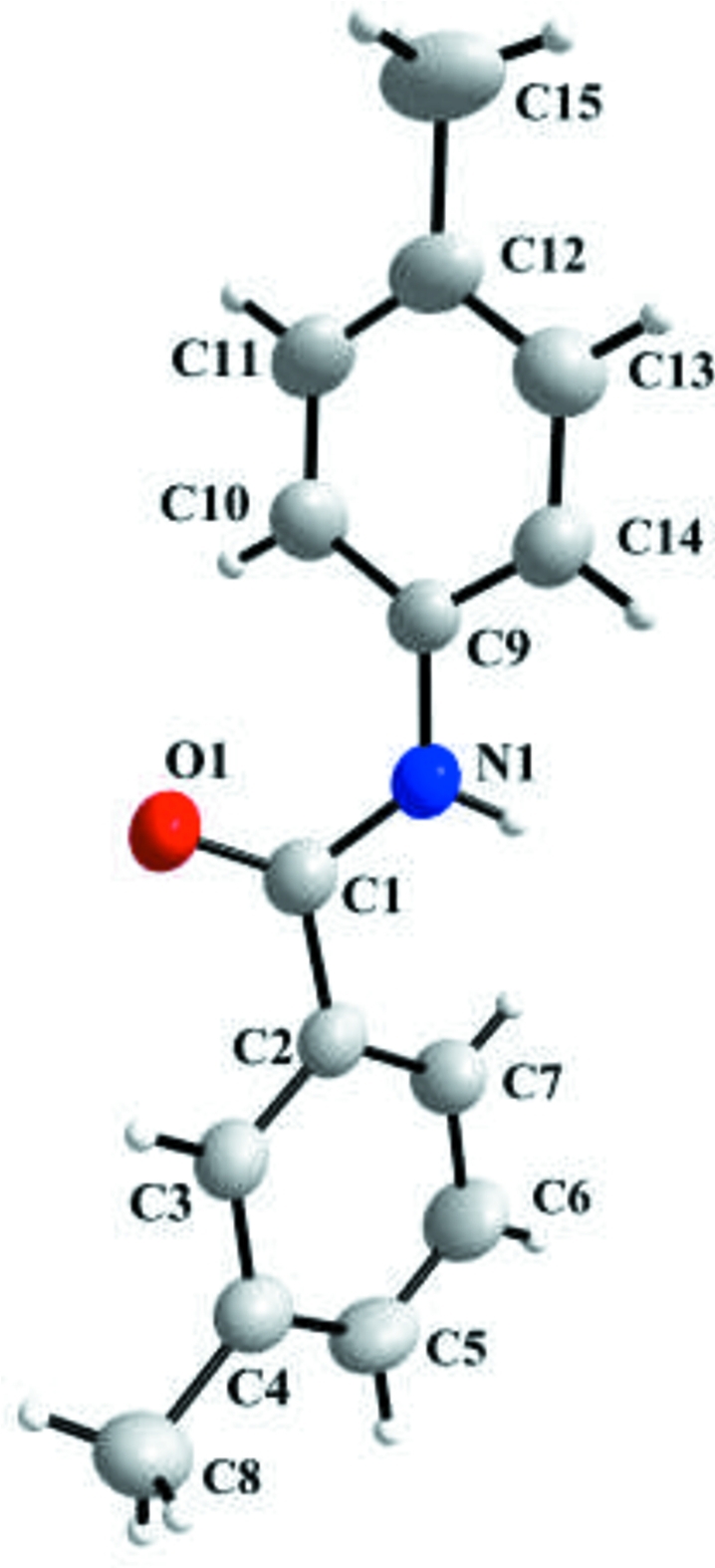
Molecular structure of the title compound showing the atom labelling scheme. Displacement ellipsoids are drawn at the 50% probability level.

**Fig. 2.**
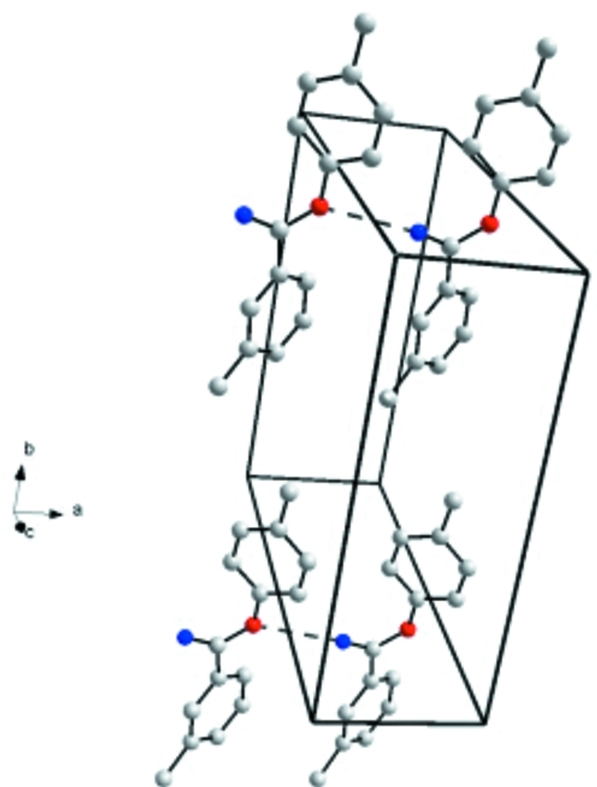
Part of the crystal structure of the title compound. Molecular chains are generated by N—H···O hydrogen bonds which are shown by dashed lines. H atoms not involved in intermolecular bonding have been omitted.

## supplementary crystallographic information

### Comment

The amide and sulfonamide moieties are the constituents of many biologically significant compounds. As part of our studies on the substituent effects on the structures and other aspects of *N*-(aryl)-amides (Bowes *et al.*, 2003; Gowda *et al.*, 2000; Saeed *et al.*, 2010), *N*-(aryl)-methanesulfonamides (Gowda *et al.*, 2007), *N*-(aryl)-arylsulfonamides (Shetty & Gowda, 2005) and *N*-chloro-arylsulfonamides (Gowda & Shetty, 2004), in the present work, the crystal structure of 3-methyl-N-(4-methylphenyl)benzamide (I) has been determined (Fig.1).

In (I), the two aromatic rings make the dihedral angle of 70.06 (3)°, while the central amide core –NH—C(=O)– is twisted by 30.24 (4) ° and 40.16 (3)° out of the planes of the 3-methyphenyl and 4-methyphenyl rings, respectively. The methyl groups are disordered over two equally occupied positions.

Further, the *meta*-methyl group in the benzoyl ring is positioned *syn* to the C=O bond, while the N—H and C=O bonds in the C—NH—C(O)—C segment are *anti* to each other.

In the crystal structure, intermolecular N—H···O hydrogen bonds link the molecules into infinite chains running along the *a*-axis. Part of the crystal structure is shown in Fig. 2.

### Experimental

The title compound was prepared according to the method described by Gowda *et al.* (2003). The purity of the compound was checked by determining its melting point. It was characterized by recording its infrared and NMR spectra. Plate like colourless single crystals of the title compound were obtained by slow evaporation of an ethanol solution of the compound (0.5 g in about 30 ml of ethanol) at room temperature.

### Refinement

All hydrogen atoms except amide H atom were placed in calculated positions with C–H distances of 0.93 Å (C-aromatic), 0.96 Å (*C*-methyl) and constrained to ride on their parent atoms. The methyl groups of the aromatic ring are disordered over two equally occupied positions rotated with respect to each other by 60°. The amide H atom was seen in difference map and was refined with the N—H distance restrained to 0.86 (2) Å. The *U*_iso_(H) values were set at 1.2 *U*_eq_(C-aromatic, N) and 1.5 *U*_eq_(*C*-methyl).

### Crystal data

**Table:**

C_15_H_15_NO	*F*(000) = 480
*M**_r_* = 225.28	*D*_x_ = 1.218 Mg m^−^^3^
Monoclinic, *P*2_1_/*c*	Mo *K*α radiation, λ = 0.71073 Å
*a* = 5.2694 (2) Å	Cell parameters from 3030 reflections
*b* = 14.0877 (5) Å	θ = 3.5–28.9°
*c* = 16.5665 (9) Å	µ = 0.08 mm^−^^1^
β = 92.588 (4)°	*T* = 295 K
*V* = 1228.54 (9) Å^3^	Plate, colourless
*Z* = 4	0.32 × 0.21 × 0.12 mm

### Data collection

**Table:**

Oxford Diffraction Xcalibur diffractometer	2800 independent reflections
Radiation source: fine-focus sealed tube	1530 reflections with *I* > 2σ(*I*)
graphite	*R*_int_ = 0.037
Detector resolution: 0 pixels mm^-1^	θ_max_ = 27.5°, θ_min_ = 2.5°
ω scans with κ offsets	*h* = −6→6
Absorption correction: multi-scan (*CrysAlis RED*; Oxford Diffraction, 2009)	*k* = −17→18
*T*_min_ = 0.976, *T*_max_ = 0.989	*l* = −21→21
18822 measured reflections	

### Refinement

**Table:**

Refinement on *F*^2^	Primary atom site location: structure-invariant direct methods
Least-squares matrix: full	Secondary atom site location: difference Fourier map
*R*[*F*^2^ > 2σ(*F*^2^)] = 0.036	Hydrogen site location: inferred from neighbouring sites
*wR*(*F*^2^) = 0.088	H atoms treated by a mixture of independent and constrained refinement
*S* = 0.83	*w* = 1/[σ^2^(*F*_o_^2^) + (0.0511*P*)^2^] where *P* = (*F*_o_^2^ + 2*F*_c_^2^)/3
2800 reflections	(Δ/σ)_max_ < 0.001
158 parameters	Δρ_max_ = 0.13 e Å^−^^3^
1 restraint	Δρ_min_ = −0.15 e Å^−^^3^

### Special details

**Table:**

Geometry. All e.s.d.'s (except the e.s.d. in the dihedral angle between two l.s. planes) are estimated using the full covariance matrix. The cell e.s.d.'s are taken into account individually in the estimation of e.s.d.'s in distances, angles and torsion angles; correlations between e.s.d.'s in cell parameters are only used when they are defined by crystal symmetry. An approximate (isotropic) treatment of cell e.s.d.'s is used for estimating e.s.d.'s involving l.s. planes.
Refinement. Refinement of *F*^2^ against ALL reflections. The weighted *R*-factor *wR* and goodness of fit *S* are based on *F*^2^, conventional *R*-factors *R* are based on *F*, with *F* set to zero for negative *F*^2^. The threshold expression of *F*^2^ > σ(*F*^2^) is used only for calculating *R*-factors(gt) *etc*. and is not relevant to the choice of reflections for refinement. *R*-factors based on *F*^2^ are statistically about twice as large as those based on *F*, and *R*- factors based on ALL data will be even larger.

### Fractional atomic coordinates and isotropic or equivalent isotropic displacement parameters (Å^2^)

**Table:**

	*x*	*y*	*z*	*U*_iso_*/*U*_eq_	Occ. (<1)
C1	0.6296 (2)	0.39571 (9)	0.60497 (7)	0.0465 (3)	
C2	0.6781 (2)	0.29397 (8)	0.58628 (7)	0.0440 (3)	
C3	0.5224 (2)	0.22617 (9)	0.61906 (7)	0.0485 (3)	
H3	0.3922	0.2461	0.6511	0.058*	
C4	0.5536 (2)	0.12994 (9)	0.60585 (8)	0.0522 (3)	
C5	0.7430 (2)	0.10317 (10)	0.55521 (8)	0.0611 (4)	
H5	0.7662	0.0391	0.5440	0.073*	
C6	0.8977 (3)	0.16938 (11)	0.52114 (9)	0.0633 (4)	
H6	1.0228	0.1495	0.4871	0.076*	
C7	0.8694 (2)	0.26472 (10)	0.53688 (8)	0.0532 (3)	
H7	0.9771	0.3090	0.5147	0.064*	
C8	0.3925 (3)	0.05761 (11)	0.64662 (10)	0.0733 (5)	
H8A	0.2724	0.0895	0.6790	0.110*	0.50
H8B	0.4992	0.0174	0.6804	0.110*	0.50
H8C	0.3032	0.0198	0.6064	0.110*	0.50
H8D	0.4441	−0.0050	0.6315	0.110*	0.50
H8E	0.2173	0.0670	0.6302	0.110*	0.50
H8F	0.4134	0.0647	0.7042	0.110*	0.50
C9	0.8328 (2)	0.55183 (9)	0.62876 (7)	0.0437 (3)	
C10	0.6436 (2)	0.61320 (10)	0.60252 (8)	0.0547 (4)	
H10	0.5077	0.5911	0.5700	0.066*	
C11	0.6557 (2)	0.70751 (10)	0.62446 (9)	0.0592 (4)	
H11	0.5264	0.7481	0.6062	0.071*	
C12	0.8528 (2)	0.74372 (9)	0.67253 (8)	0.0537 (3)	
C13	1.0412 (3)	0.68103 (10)	0.69753 (9)	0.0620 (4)	
H13	1.1777	0.7031	0.7298	0.074*	
C14	1.0328 (2)	0.58675 (10)	0.67604 (8)	0.0568 (4)	
H14	1.1634	0.5463	0.6936	0.068*	
C15	0.8641 (3)	0.84701 (10)	0.69657 (10)	0.0796 (5)	
H15A	1.0149	0.8583	0.7299	0.119*	0.50
H15B	0.7173	0.8627	0.7262	0.119*	0.50
H15C	0.8673	0.8858	0.6490	0.119*	0.50
H15D	0.7181	0.8795	0.6734	0.119*	0.50
H15E	1.0157	0.8751	0.6772	0.119*	0.50
H15F	0.8657	0.8521	0.7544	0.119*	0.50
N1	0.83366 (19)	0.45397 (8)	0.60708 (6)	0.0491 (3)	
O1	0.41508 (15)	0.42350 (6)	0.61856 (6)	0.0643 (3)	
H1	0.980 (2)	0.4272 (8)	0.6061 (7)	0.049 (4)*	

### Atomic displacement parameters (Å^2^)

**Table:**

	*U*^11^	*U*^22^	*U*^33^	*U*^12^	*U*^13^	*U*^23^
C1	0.0427 (7)	0.0474 (8)	0.0496 (8)	0.0044 (6)	0.0048 (5)	−0.0005 (6)
C2	0.0402 (6)	0.0455 (8)	0.0461 (7)	0.0052 (6)	−0.0012 (5)	−0.0019 (6)
C3	0.0433 (6)	0.0493 (9)	0.0530 (8)	0.0046 (6)	0.0034 (5)	−0.0028 (6)
C4	0.0513 (7)	0.0481 (9)	0.0564 (8)	0.0021 (6)	−0.0055 (6)	0.0010 (6)
C5	0.0685 (8)	0.0458 (8)	0.0686 (10)	0.0087 (7)	−0.0016 (7)	−0.0068 (7)
C6	0.0652 (8)	0.0600 (10)	0.0659 (9)	0.0136 (8)	0.0167 (7)	−0.0071 (8)
C7	0.0529 (7)	0.0503 (9)	0.0570 (8)	0.0034 (6)	0.0099 (6)	−0.0014 (7)
C8	0.0740 (9)	0.0552 (10)	0.0908 (12)	−0.0059 (7)	0.0041 (8)	0.0095 (8)
C9	0.0416 (6)	0.0416 (8)	0.0485 (7)	0.0016 (6)	0.0070 (5)	0.0010 (6)
C10	0.0492 (7)	0.0506 (9)	0.0636 (9)	0.0015 (6)	−0.0054 (6)	0.0062 (7)
C11	0.0560 (8)	0.0475 (9)	0.0742 (10)	0.0114 (7)	0.0036 (7)	0.0106 (7)
C12	0.0633 (8)	0.0447 (8)	0.0543 (8)	0.0025 (6)	0.0156 (7)	−0.0011 (6)
C13	0.0612 (8)	0.0569 (10)	0.0670 (9)	−0.0020 (7)	−0.0061 (7)	−0.0104 (7)
C14	0.0485 (7)	0.0496 (9)	0.0717 (9)	0.0087 (6)	−0.0047 (6)	−0.0045 (7)
C15	0.1069 (12)	0.0512 (10)	0.0821 (11)	0.0041 (9)	0.0190 (9)	−0.0072 (8)
N1	0.0377 (5)	0.0447 (7)	0.0653 (7)	0.0049 (5)	0.0059 (5)	−0.0027 (5)
O1	0.0427 (5)	0.0512 (6)	0.1003 (7)	0.0044 (4)	0.0162 (4)	−0.0083 (5)

### Geometric parameters (Å, °)

**Table:**

C1—O1	1.2267 (12)	C9—C14	1.3752 (17)
C1—N1	1.3519 (15)	C9—C10	1.3754 (16)
C1—C2	1.4908 (17)	C9—N1	1.4247 (16)
C2—C3	1.3855 (17)	C10—C11	1.3782 (18)
C2—C7	1.3894 (16)	C10—H10	0.9300
C3—C4	1.3843 (18)	C11—C12	1.3776 (18)
C3—H3	0.9300	C11—H11	0.9300
C4—C5	1.3849 (18)	C12—C13	1.3783 (18)
C4—C8	1.5056 (19)	C12—C15	1.5091 (19)
C5—C6	1.3764 (19)	C13—C14	1.3753 (19)
C5—H5	0.9300	C13—H13	0.9300
C6—C7	1.3776 (19)	C14—H14	0.9300
C6—H6	0.9300	C15—H15A	0.9600
C7—H7	0.9300	C15—H15B	0.9600
C8—H8A	0.9600	C15—H15C	0.9600
C8—H8B	0.9600	C15—H15D	0.9600
C8—H8C	0.9600	C15—H15E	0.9600
C8—H8D	0.9600	C15—H15F	0.9600
C8—H8E	0.9600	N1—H1	0.857 (10)
C8—H8F	0.9600		
			
O1—C1—N1	122.68 (12)	C14—C9—C10	118.76 (12)
O1—C1—C2	120.86 (11)	C14—C9—N1	118.60 (11)
N1—C1—C2	116.46 (10)	C10—C9—N1	122.62 (11)
C3—C2—C7	118.98 (12)	C9—C10—C11	119.89 (12)
C3—C2—C1	118.12 (10)	C9—C10—H10	120.1
C7—C2—C1	122.89 (11)	C11—C10—H10	120.1
C4—C3—C2	122.45 (11)	C12—C11—C10	122.29 (12)
C4—C3—H3	118.8	C12—C11—H11	118.9
C2—C3—H3	118.8	C10—C11—H11	118.9
C3—C4—C5	117.15 (12)	C11—C12—C13	116.76 (13)
C3—C4—C8	121.24 (12)	C11—C12—C15	121.96 (13)
C5—C4—C8	121.59 (13)	C13—C12—C15	121.28 (13)
C6—C5—C4	121.34 (13)	C14—C13—C12	121.79 (13)
C6—C5—H5	119.3	C14—C13—H13	119.1
C4—C5—H5	119.3	C12—C13—H13	119.1
C5—C6—C7	120.80 (12)	C9—C14—C13	120.51 (12)
C5—C6—H6	119.6	C9—C14—H14	119.7
C7—C6—H6	119.6	C13—C14—H14	119.7
C6—C7—C2	119.22 (12)	C12—C15—H15A	109.5
C6—C7—H7	120.4	C12—C15—H15B	109.5
C2—C7—H7	120.4	H15A—C15—H15B	109.5
C4—C8—H8A	109.5	C12—C15—H15C	109.5
C4—C8—H8B	109.5	H15A—C15—H15C	109.5
H8A—C8—H8B	109.5	H15B—C15—H15C	109.5
C4—C8—H8C	109.5	C12—C15—H15D	109.5
H8A—C8—H8C	109.5	H15A—C15—H15D	141.1
H8B—C8—H8C	109.5	H15B—C15—H15D	56.3
C4—C8—H8D	109.5	H15C—C15—H15D	56.3
H8A—C8—H8D	141.1	C12—C15—H15E	109.5
H8B—C8—H8D	56.3	H15A—C15—H15E	56.3
H8C—C8—H8D	56.3	H15B—C15—H15E	141.1
C4—C8—H8E	109.5	H15C—C15—H15E	56.3
H8A—C8—H8E	56.3	H15D—C15—H15E	109.5
H8B—C8—H8E	141.1	C12—C15—H15F	109.5
H8C—C8—H8E	56.3	H15A—C15—H15F	56.3
H8D—C8—H8E	109.5	H15B—C15—H15F	56.3
C4—C8—H8F	109.5	H15C—C15—H15F	141.1
H8A—C8—H8F	56.3	H15D—C15—H15F	109.5
H8B—C8—H8F	56.3	H15E—C15—H15F	109.5
H8C—C8—H8F	141.1	C1—N1—C9	125.62 (10)
H8D—C8—H8F	109.5	C1—N1—H1	116.5 (8)
H8E—C8—H8F	109.5	C9—N1—H1	116.3 (8)
			
O1—C1—C2—C3	−29.29 (17)	C14—C9—C10—C11	−0.67 (18)
N1—C1—C2—C3	149.82 (11)	N1—C9—C10—C11	−178.89 (12)
O1—C1—C2—C7	149.86 (12)	C9—C10—C11—C12	−0.1 (2)
N1—C1—C2—C7	−31.03 (17)	C10—C11—C12—C13	0.58 (19)
C7—C2—C3—C4	1.41 (18)	C10—C11—C12—C15	−179.75 (13)
C1—C2—C3—C4	−179.40 (11)	C11—C12—C13—C14	−0.4 (2)
C2—C3—C4—C5	−2.49 (19)	C15—C12—C13—C14	179.96 (13)
C2—C3—C4—C8	175.97 (11)	C10—C9—C14—C13	0.89 (19)
C3—C4—C5—C6	1.58 (19)	N1—C9—C14—C13	179.17 (12)
C8—C4—C5—C6	−176.87 (13)	C12—C13—C14—C9	−0.4 (2)
C4—C5—C6—C7	0.4 (2)	O1—C1—N1—C9	3.39 (19)
C5—C6—C7—C2	−1.5 (2)	C2—C1—N1—C9	−175.70 (11)
C3—C2—C7—C6	0.64 (18)	C14—C9—N1—C1	139.30 (12)
C1—C2—C7—C6	−178.50 (11)	C10—C9—N1—C1	−42.48 (18)

### Hydrogen-bond geometry (Å, °)

**Table:**

*D*—H···*A*	*D*—H	H···*A*	*D*···*A*	*D*—H···*A*
N1—H1···O1^i^	0.86 (1)	2.30 (1)	3.0910 (13)	155.(1)

Symmetry codes: (i) *x*+1, *y*, *z*.
